# Inflammatory Mediators in Oral Cancer: Pathogenic Mechanisms and Diagnostic Potential

**DOI:** 10.3389/froh.2021.642238

**Published:** 2021-02-22

**Authors:** Sven E. Niklander

**Affiliations:** Unidad de Patologia y Medicina Oral, Facultad de Odontologia, Universidad Andres Bello, Viña del Mar, Chile

**Keywords:** oral cancer, inflammation, oral carcinogenesis, biomarker, OPMD

## Abstract

Approximately 15% of cancers are attributable to the inflammatory process, and growing evidence supports an association between oral squamous cell carcinoma (OSCC) and chronic inflammation. Different oral inflammatory conditions, such as oral lichen planus (OLP), submucous fibrosis, and oral discoid lupus, are all predisposing for the development of OSCC. The microenvironment of these conditions contains various transcription factors and inflammatory mediators with the ability to induce proliferation, epithelial-to-mesenchymal transition (EMT), and invasion of genetically predisposed lesions, thereby promoting tumor development. In this review, we will focus on the main inflammatory molecules and transcription factors activated in OSCC, with emphasis on their translational potential.

## Introduction

The links between cancer and chronic inflammation are well-established. In this process, pro-inflammatory mediators act by stimulating inflammation to either abrogate tumor progression or facilitate tumor growth and metastasis [1, 2]. Approximately 15% of cancers are attributable to the inflammatory process [3]. Such cancers include lung, pancreatic, esophageal, bladder, gastric, cervical, colorectal, and prostate [4]. During the oral malignant transformation process [from non-dysplastic hyperkeratosis, through oral dysplasia to the development of oral squamous cell carcinoma (OSCC)], there is a progressive increase of the inflammatory infiltrate (quality and density) [5], and growing evidence supports an association between OSCC and chronic inflammation [6, 7]. Different oral inflammatory conditions, such as OLP, submucous fibrosis, and oral discoid lupus, are all predisposing for the development of OSCC [8]. The microenvironment of these conditions contains activated cytokines and chemokines, prostaglandins, reactive oxygen species, and various transcription factors. Some of these mediators have the ability to induce proliferation, epithelial-to-mesenchymal transition (EMT), and invasion [9] of genetically predisposed lesions (with mutations in tumor-suppressor genes and/or oncogenes), thereby promoting tumor development. In established OSCC, chronic inflammation is also a common feature, being involved in tumor progression, invasion, and metastasis [5, 6, 10]. Thus, many studies have assessed the utility of different inflammatory molecules as prognostic biomarkers and treatment targets for OSCC.

The aim of this review is to outline the main inflammatory molecules and transcription factors activated in OSCC, with special focus on their translational potential.

## Inflammatory Mediators Involved in Oral Carcinogenesis

There are different inflammatory mediators reported to have a role in the development and progression of OSCC (Table 1). In this review, we will focus on the most commonly investigated.

**Table 1:** Inflammatory pathways and mediators reported to have a role in oral carcinogenesis.

**Pro-inflammatory cytokines**	**References**
IL-1	[7, 9, 11, 12]
IL-1R1	[13]
IL-6	[6, 11, 14]
IL-8	[6, 11, 14, 15]
TNF-α	[5, 11, 16]
TGF-β	[17–19]
**Immunosuppresive Cytokines**	
IL-1RA	[20, 21]
IL-2	[22]
IL-4	[22, 23]
IL-10	[24–26]
IL-12	[27]
IL-13	[24]
IFN-γ	[14, 28]
**Chemokines**	
CCL	[29]
CXCL1	[30]
CXCL2	[29, 31]
CCL4	[32]
CCL7	[33]
CXCL3	[29]
CXCL10	[34]
CXCL12	[31]
CXCR1	[35, 36]
CXCR2	[36]
**Transcription factros**	
AP-1	[37–39]
NF-kB	[6, 40–42]
**Enzymes**	
COX2	[43–45]
**Prostanoids**	
PGE2	[46]

### NF-κB

Nuclear factor kappa-beta (NF-κB) is a key inflammatory transcription factor frequently expressed in tumors that regulates the expression of a variety of genes involved in inflammation, proliferation, tumorigenesis, and cell survival [47–49]. NF-κB is canonically activated by tumor necrosis factor alpha (TNF-α), interleukin (IL)-1, and lipopolysaccharide (LPS) [48], and when activated, it enhances the expression of different cytokines, including IL-1, IL-6, and IL-8 [50]. Its aberrant expression is linked to carcinogenesis [51] and EMT induction [52] and is associated with worse survival in solid cancers [53]. In OSCC, NF-κB is constitutively activated and is associated with the upregulation of different inflammatory genes, including *IL-6, IL-8, CCL5*, and *CXCL10* [6], and is considered a major factor responsible for the inflammatory infiltrate observed in the tumor microenvironment (TME) [54]. NF-κB has an important role in the malignant phenotype of oral cancers, as it participates in the modulation of bone invasion [48]; enhances angiogenesis [55], invasion [40, 56], and metastasis [40, 41]; and induces EMT [52]. The EMT process is a crucial step for the development of OSCC metastasis [57]. It involves the repression of E-cadherin (an important epithelial adhesion molecule) *via* Snail expression, which depends on NF-κB activation *via* AKT [52]. IL-8 and EGF are able to induce EMT *via* NF-κB activation, and IL-8- and EGF-induced EMT can be reversed by blocking AKT or NF-κB [58, 59]. NF-κB importance during OSCC development is well-exemplified by its inactivation, as this inhibits cell survival and growth; expressions of IL-1α, IL-6, and IL-8 [60]; and metastases [41]. Because of this, NF-κB inhibition has been proposed as a possible treatment for head and neck squamous cell carcinoma (HNSCC).

### AP-1 Pathway

Activator protein 1 (AP-1) is a transcription factor complex composed of either homodimers of Jun protein or heterodimers of Jun and Fos proteins [61] that orchestrates the expression of different genes involved in inflammation, embryonic development, lymphoid proliferation, oncogenesis, and apoptosis [62] and is reported to be essential for DNA synthesis [63]. AP-1 activation seems to be of clinical significance in cancer, as high expression levels have been associated with drug resistance [64]. AP-1 is activated during oral keratinocyte carcinogenesis [37], and its expression increases with oral tumor progression [38]. Similar to NF-κB, it can also be activated by IL-1 [65], which induces IL-8 secretion and promotes the cell survival and growth of HNSCC cells [39]. A recent study suggested that AP-1 induces bcl-2 expression (a proto-oncogene related to apoptosis suppression implicated in resistance to chemoradiation therapy in OSCC) in recurrent chemo- and radioresistant oral tumors [38], which suggests that targeting the AP-1 pathway might contribute to overcome resistance to chemoradiation therapy in OSCC. As AP-1 can be activated by IL-1, targeting IL-1 could be beneficial for oral cancer treatment, as this could reduce the activation of NF-κB and AP-1 pathways with subsequent reduction of bcl-2, but this hypothesis needs to be corroborated.

### TNF-α

TNF-α is a multifunctional cytokine identified as an important mediator of cancer development [66], as well as induces EMT [67] and enhances tumor angiogenesis [68] and invasion [56]. Therefore, it is considered an important regulator of proliferation, invasion, and metastasis of many cancers [69]. TNF-α is expressed in some oral potentially malignant disorders (OPMDs), such as OLP [70], and is endogenously expressed in oral carcinomas [71]. In OSCC, TNF-α promotes a pro-invasive and pro-inflammatory phenotype in a paracrine manner [5, 16] by upregulating genes that are associated with neutrophil recruitment, invadopodia, and invasion. The upregulation of these genes was also associated with reductions in both the overall survival and disease-free survival of patients with OSCC [5]. Elevated TNF-α receptor-1 (TNFR-1) signaling has also been associated with metastasis of OSCC [41, 72], attributable to the ability of TNF-α to stimulate the invasion of OSCC cells by enhancing matrix metalloproteinase (MMP)-2 and MMP-9 production [41, 56], which is regulated by the NF-κB, AKT, and PI3K signaling pathways [5, 41, 72]. It has been demonstrated that MMPs play critical roles in OSCC, as elevated expression of MMPs is associated with increased OSCC invasion, metastasis, and poor prognosis [73–75]. MMPs are also reported to have a prominent role in the EMT process [76], as MMP-2, MMP-9, and MMP-7 are able to enhance EMT [77–79]. NF-κB is an important regulator of MMPs [80], and TNF-α can activate NF-κB *via* TNFR1, which results in enhanced MMP secretion [81]. TNF-α is also able to induce EMT *via* p38 MAPK activation [82], and TNF-α-induced EMT has been related to the induction of cancer stem cells (CSCs) [67]. The presence of CSCs in OSCC is of importance as this has been linked to therapy resistance and worst prognosis. In OSCC, the expression of CD44 (a well-known CSC marker) has been associated with increased cell invasion, cell migration, and therapy resistance *via* a mechanism that includes the activation of PI3K/Akt/GSK3β and Raf-MEK-ERK signaling networks [83], and silencing CD47 (a molecule involved in the generation of CSCs in OSCC) has shown to reduce EMT and the presence of CSCs [84]. The activation of the PI3K/Akt/GSK3β signaling pathway has also been associated with increased proliferation, invasion [85], the development of EMT, and distant metastasis [86].

Due to all the beneficial effects of TNF-α during cancer development, targeting TNF-α might be a useful strategy for OSCC treatment. *In vitro* experiments have shown anti-TNF-α therapy to reduce growth and metastasis of OSCC cells [87], but this needs to be investigated further before it can be translated into the clinic.

### IL-6 and IL-8

Both IL-6 and IL-8 are considered “oncogenic cytokines,” as they are able to cause EMT [88], stimulate angiogenesis and tumor growth [89, 90], disrupt cell–cell communication, impede macrophage function, and promote epithelial and endothelial cell migration and invasion [91]. IL-6 and IL-8 levels are elevated in patients with OPMDs and OSCCs [6, 14, 92, 93], which is likely to be a consequence of aberrant NF-κB activation [94]. IL-6 and IL-8 can be produced by malignant oral keratinocytes themselves, or by other cells of the TME, such as tumor-associated macrophages (TAMs). TAMs are an important source of both of these cytokines, and thus many attempts have been done to target TAMs to restrict their secretion [95]. IL-8 is reported to act as an autocrine growth factor in HNSCC and other cancers [15] and has been proposed as a potential mediator of the development of OSCC. It is constitutively expressed in malignant oral keratinocytes, and its inhibition decreases viability, proliferation [6], and invasion of OSCC cells [16] and enhances the proliferation, angiogenesis, and survival rate of cancer cells [96]. Similarly, IL-6 overexpression in patients with HNSCC is associated with poor prognosis, probably by enabling an immunosuppressive TME by increasing the presence of myeloid-derived suppressor cells and PDL-1 expression, and is considered a significant predictor of treatment outcome [97]. In OSCC, the expressions of IL-6 and IL-8 are associated with a more invasive mode of growth [11].

### IL-1 Family Members

IL-1 is the prototype of a pro-inflammatory cytokine and includes IL-1α and IL-1β. IL-1α and IL-1β are both constitutively expressed in OSCC [9, 12, 98, 99], can be found in the saliva of patients with OSCC [100, 101], and have been reported to have important functions in OSCC carcinogenesis and tumor progression [7, 10].

IL-1α expressed by OSCC promotes autocrine activation of NF-κB and AP-1 and upregulates the expression of IL-8 [39] and IL-6 [98]. OSCC cells produce IL-1α [12], which induces the proliferation and cytokine secretion by cancer-associated fibroblasts (CAFs) (CCL7, CXCL1, and IL-8), promoting tumor progression [33]. IL-1α seems to be important for the development of distant metastases, as IL-1α is highly expressed in metastatic HNSCC tumors compared to non-metastatic HNSCC tumors. This is probably achieved by the capability of IL-1α to induce transmigration of tumor cells across the endothelium and to enhance the expression of metastatic genes, such as *MMP-*9, *PGE2, VEGF*, and *IL-8* [10]. It has also been reported that IL-1α can act as an oncoprotein by itself, as intranuclear IL-1α has been shown to induce malignant transformation of cells from the bone marrow and perivascular area [102].

IL-1β increases the levels of IL-6 and IL-8 expressed by OD and OSCC cells and promotes the invasiveness of OSCC by inducing EMT [9]. IL-1β has also been identified as a key node gene in the TME of OSCC *in vivo* [7]. The expression of precursor IL-1β mRNA is correlated with the presence of malignant changes (from normal, to mild, through severe dysplasia to OSCC) [9], and elevated IL-1β expression has been related to lymph node metastasis of OSCC [103]. IL-1 produced by HNSCC can also stimulate the production of different cytokines by CAFs and normal fibroblasts, such as CCL-7, CXCL1, IL-8, and CCL-5 [33, 104]. These findings strongly suggest a possible role of IL-1β in the oral carcinogenesis process, which is supported by the fact that IL-1β silencing can reduce tumor size *in vivo* [7].

The IL-1 agonist receptor, IL-1R1, is also overexpressed in OSCCs and, together with IL-1β, was shown to promote cancer growth and metastasis by upregulating CXCR4, which could be reversed by inhibiting IL-1R1 by overexpressing the interleukin 1 receptor antagonist (IL-1RA) [13]. Interestingly, IL-1RA has been reported to be downregulated in OD and in OSCC [20] and is reported to regulate IL-1-induced secretion of IL-6 and IL-8 by inhibiting the p38 MAPK and NF-κB pathways [50, 105].

### COX-2

Cyclooxygenase (COX)-2, an inflammation-induced enzyme that converts arachidonic acid into prostaglandins [e.g., prostaglandin E2 (PGE2)], is frequently expressed in many types of cancers. COX-2 is able to induce CSC-like activity and to promote angiogenesis, proliferation, apoptotic resistance, inflammation, invasion, and metastasis of cancer cells [106]. Importantly, COX-2 inhibition has been shown to reverse cancer progression [107, 108]. COX-2 is induced by a variety of molecules, including IL-1 [109], epithelial growth factor (EGF) [110], transforming growth factor-beta (TGF-β) [111, 112], and TNF-α. COX-2 expression is induced early in the process of oral carcinogenesis [43]. Its level is associated with the degree of dysplasia [44], is overexpressed in OSCC [113, 114], and is correlated with advanced tumor stage, high risk of distant metastasis [115], and worse prognosis in patients with OSCC [116]. In oral cancer, COX-2 is of importance for maintaining a chronic inflammatory state [117], influencing different processes, such as cell migration by upregulating the expression of intercellular adhesion molecule-1 (ICAM-1) *via* PGE2 [46], and lymphoangiogenesis by regulating VEGF production [45, 118, 119]. VEGF is commonly overexpressed in OSCC, and COX-2/VEGF-C co-expression is correlated with lymphoangiogenesis, lymph node metastasis, and TNM stage and is reported as an independent factor for survival [45]. Increased VEGF expression in oral cancer is also a consequence of tumor-associated hypoxia, as VEGF is upregulated in decreasing concentrations of oxygen [120, 121]. In reduced oxygen concentrations, hypoxia-inducible factor-1α (HIF-1α) binds to hypoxia response elements and upregulates *VEGF*, promoting angiogenesis [122]. In OSCC, HIF-1α and HIF-2α correlate positively with clinical–pathological parameters, such as tumor size and micro vessel density, and *in vivo* experiments have shown their knockdown to reduce tumor angiogenesis and tumor growth [123]. In addition, hypoxia will also promote an inflammatory state, as VEGF is able to induce COX-2 expression, which will result in the production of PGE2 and the activation of NF-κB [124].

### TGF-β

TGF-β is a multifunctional pro-inflammatory cytokine that can either inhibit or promote tumor formation and progression of many cancers, whether by inducing apoptosis and growth arrest and by inhibiting proliferation or by stimulating angiogenesis, inflammation, EMT, and immune suppression, respectively [125]. If TGF-β acts as a tumor suppressor or tumor promoter depends on the regional and cellular context [126]. In OSCC, TGF-β is reported to promote tumorigenesis [17]. This is supported by the fact that OPMDs [18] and OSCC [19, 127] express higher levels of TGF-β than healthy controls and that high TGF-β levels are associated with disease recurrence and poor prognosis in patients with OSCC [19]. There are different mechanisms by which TGF-β could act as a tumor promoter in oral cancer. TGF-β is probably the most important factor involved in the differentiation of CAFs, and the accumulation of CAFs is reported as an independent prognostic factor in OSCC. CAFs are able to modulate the TME, facilitating cancer progression [128]. An *in vivo* mouse model in which TGF-β1 was transgenically induced revealed that TGF-β1 induced epithelia hyperproliferation, severe inflammation, and angiogenesis at similar levels to those observed in HNSCCs, suggesting that TGF-β1 provides a tumor-promoting microenvironment [17]. In OPMDs, TGF-β promotes a more malignant phenotype by increasing cell motility *via* the protein phosphatase 1 (PP-1) signaling pathway [129]. TGF-β seems to be important for EMT development, as TGF-β induces EMT in endothelial cells, and endothelial cells cultured with TGF-β are able to induce EMT in OSCC cells [130]. Also, TGF-β is able to induce EMT by stimulating the expression of ADAM12 (a desintegrin and metalloprotease associated with cancers) [131].

### Immunosuppressive Cytokines

Anti-inflammatory cytokines also have a role in the oral carcinogenesis process. They can act as a double-edged sword, they can counteract the tumorigenic potential of their pro-inflammatory counterpart, and they can act as immunosuppressive molecules by decreasing the anti-tumor immune response [132]. Different immunosuppressive cytokines have been reported to have different roles in OSCC development (Table 1), with IL-1RA, IL-4, IL-10, and IL-13 being the most commonly investigated. IL-1RA decreases during the oral carcinogenesis process [20] and *IL1RN* (the gene that codes for IL-1RA) is downregulated in HNSCC [133, 134]. This in theory would allow higher IL-1 activity with the aforementioned effects. Nevertheless, high IL1-RA expression has been reported in advanced and poorly differentiated OSCCs [20, 24], suggesting that IL-1RA expression could increase tumor progression, but this needs further investigation. IL-4, IL-10, and IL-13 have also been reported to increase in OSCC patients compared to healthy controls [24, 25, 135], and high IL-10 expression has been associated with a more aggressive OSCC phenotype [136]. IL-4 induces immune deviation from T_H_1 to T_H_2 responses, which prevents tumor rejection [137]; IL-10 suppresses the anti-tumor immunity and contributes to tumor immune escape [135] and IL-13 compromises the anti-tumor response by inhibiting IFN-γ secretion and CD8+ T lymphocyte activity [138].

## Sources of Inflammation

Within the TME, cancer cells are not the only source of inflammatory molecules. Tumors are complex systems composed not only of neoplastic cells but also of stromal cells, which form the TME. These cells are not innocent bystanders and can interact with tumor cells and modify the extracellular matrix, facilitating and promoting proliferation, invasion, angiogenesis, and metastasis [49, 139]. Single cell profile of 5,578 samples obtained from 18 OSCCs revealed the presence of nine clusters of cells based on known marker genes, such as epithelial cells (malignant and non-malignant, based on CNV and their karyotypes), T cells (four sub-clusters), B/plasma cells, macrophages, dendritic cells, mast cells, endothelial cells, fibroblasts (three sub-clusters), and myocytes [140], which shows how heterogeneous tumors are.

As discussed previously, malignant oral keratinocytes (which are genetically unstable) activate different transcription factors, such as AP-1 and NF-κB, leading to the activation of oncogenes (which induces proliferation by the regulation of apoptosis, angiogenesis, and cell growth), and inflammatory genes [6]. The latter results in the constitutive production of different inflammatory factors, such as IL-1, IL-6, IL-8, TNF-α, and TGF-β [141], among many others, that can activate the same transcription factors, creating a positive feedback loop.

TAMs, T lymphocytes, neutrophils, and mast cells are also considered important sources of inflammatory mediators [5]. TAMs promote proliferation and invasion in OSCC [142, 143], and their presence correlates with disease progression and is considered an adverse prognostic factor [144]. They act as an important source of cytokines, metalloproteinases, and growth factors [95]. T lymphocytes are considered the most abundant inflammatory cells among the inflammatory infiltrate observed in OSCCs and can be beneficial or detrimental for oral carcinogenesis depending on their secretion profile [145]. They can secrete molecules that favor tumor progression (IL-6, IL-17, IL-23, TNF-α, and TGF-β) or molecules that exhibit an anti-tumor effect (IL-12 and IFN-γ) [49]. Neutrophil infiltration increases OSCC invasion by inducing matrix degradation and invadopodia formation through a paracrine TNF-α-induced mechanism [5, 16]. Mast cells have been associated with angiogenesis and are able to secrete numerous cytokines, chemokines, and angiogenic factors [146, 147], but their role in the development of OSCC is still debatable.

Cancer-associated fibroblasts, the most dominant components of the TME, are recipients of many of these factors (e.g., TGF-β), as well as directly contributing to tumor progression and an inflammatory TME *via* the secretion of VEGF, IL-6, IL-8, PGE2, TGF-β, and activation of NF-κB [148]. The presence and activity of CAFs are associated with poorer prognostic outcomes for OSCC [149] and are involved in bone invasion [150].

Senescent cells (which are more prevalent in older individuals, same as cancer) also contribute to chronic inflammation. When cells senesce, they develop an inflammatory secretory phenotype known as the senescent-associated secretory phenotype (SASP) [151], characterized by the secretion of high levels of “pro-oncogenic cytokines,” such as IL-1, IL-6, and IL-8 [152]. In fact, the SASP is currently recognized as a cancer promoter mechanism, as it can induce EMT, invasion, and metastasis [151, 153], and its regulation has been proposed as anti-cancer treatment [154, 155].

## Diagnostic Potential

Due to the advantages of using saliva samples (non-invasive and cost-efficient) for diagnostic and disease screening purposes, different studies have explored the possibility of using salivary inflammatory factors as biomarkers for the development and progression of OSCC. The most studied inflammatory proteins are TNF-α [5, 14, 42, 141, 156–159], IL-1 [5, 14, 141, 160, 161], IL-6 [5, 14, 34, 42, 92, 141, 158, 159, 162–164], and IL-8 [2, 5, 14, 42, 141, 160, 161, 164] (NF-κB-dependent cytokines), which have been found to be increased in the saliva of patients with different OPMDs (including OLP, oral leukoplakia, verrucous proliferative leucoplakia, and oral submucous fibrosis) and OSCC in comparison to healthy controls. Other cytokines, such as MIP-1β and IFNγ [14], and the anti-inflammatory cytokines IL-10 and IL-13 [24] have also been found to be increased in the saliva of patients with OSCC.

Using an array that analyzed the expression of 50 cytokines present in the saliva of patients with stage I OSCC before and after surgical treatment, Kamatani et al. [101] showed a significant decrease in salivary IL-1β after surgical resection. Similar to this, higher salivary levels of IL-8, IL-6, IL-1β, MIP-1β, and IP-10 before surgical intervention in patients with stage I OSCC were recently reported [165]. These results suggest that salivary inflammatory cytokines might be useful to monitor disease relapse.

A proteomic analysis of 60 saliva samples from healthy individuals and patients with OPMD and OSCC detected 21 proteins differentially expressed in OSCC compared to those in the other groups. Among those, three proteins were selected for further validation using ELISA, which included the interleukin-1 receptor antagonist (IL-1RA). Salivary IL-1RA was significantly decreased in patients with OPMD and control individuals compared to that in patients with OSCC but, alone, was suboptimal for distinguishing patients with OPMDs and healthy controls from patients with OSCC. In combination with other biomarkers (SLC3A2 and S100A2), IL-1RA was able to distinguish patients with OSCC from both healthy individuals and patients with OPMD with a sensitivity and specificity of 83.33/83.33% and of 93.33/70%, respectively [21]. Salivary IL-1β and IL-8 have also been found to discriminate between healthy individuals and patients with OSCC [160, 166] and high sensitivity and specificity values have been reported (86/97% for IL-8 and 83/76% for IL-1β) [166, 167]. Nevertheless, a recent systematic review reported average sensitivity and specificity values of 41/69% for IL-8 and of 26/46% for IL-1β [94], which are suboptimal. Both cytokines performed better when used in combination with other markers [161, 166], so it is likely that their future use will be as part of a panel of salivary biomarkers, rather than as single markers.

IL-6 is also a promising biomarker for the development of OSCC. IL-6 salivary levels are reported to increase with the severity of oral dysplasia [34], to differentiate between OPMD and OSCC (sensitivity and specificity values of 96 and 99%, respectively) [163], and to predict treatment outcomes in patients with OSCC [11, 97]. High salivary expression of TNF-α, IL-1α, and IL-8 has also been associated with a decreased survival rate in tongue squamous cell carcinoma (TSCC) [11].

## Future Direction

The understanding of the role and origin of the different inflammatory molecules involved during tumor initiation, promotion, and progression of oral cancers provides an opportunity to target inflammation to improve patient outcomes. This can be done by selectively targeting transcription pathways (e.g., NF-κB), cytokines (e.g., IL-1), or cell types known to contribute to the inflammatory secretome (e.g., CAFs and senescent cells). There are several research lines in this field, but little has been translated into the clinics, especially with regard to OSCC. Although all of the aforementioned reports are pointing in the direction that salivary cytokines could be used for diagnostic and prognostic aims in patients with oral cancer, more prospective studies are needed before they could be used in clinical settings.

## Author Contributions

SN performed the review and wrote the manuscript.

## Conflict of Interest

The author declares that the research was conducted in the absence of any commercial or financial relationships that could be construed as a potential conflict of interest.
